# Capabilities, opportunities, motivations, and practices of different sector professionals working on community environments to improve health

**DOI:** 10.17269/s41997-023-00824-y

**Published:** 2023-11-02

**Authors:** Uloma Igara Uche, Jodie Stearns, Karen Lee

**Affiliations:** Housing for Health, Division of Preventive Medicine, Faculty of Medicine and Dentistry, University of Alberta, Edmonton, AB Canada

**Keywords:** Built environment, Multi-sector professionals, Public health, COM-B, Cadre bâti, professionnels multidisciplinaires, santé publique, COM-B

## Abstract

**Objective:**

With rising healthcare costs in Canada from chronic conditions, individual behaviour change interventions in the clinical settings need to be complemented by a determinants of health approach, where multi-sector professionals assist in the creation of healthier community environments. This study sought to gain insights into capabilities, opportunities, motivations, and behaviours (COM-B) of Canadian multi-sector professionals for working together to improve built environments (BE) for health.

**Methods:**

A cross-sectional study was conducted with 61 multi-sector professionals. A 49-item questionnaire measuring constructs of COM-B for healthy BE practices was administered.

**Results:**

Public health (PH) professionals were more motivated by personal interest/values in healthy BE and the presence of scientific evidence on BE design health impacts as compared with planning and policy/program development (PPD) professionals. Planning professionals were more likely to be motivated by healthy BE legislation/regulations/codes than PPD professionals. The practice of taking responsibility for the inclusion of healthy features into BE designs was reported more often by planning and other professionals compared to PH professionals. Results trended towards significance for opportunities as a predictor of healthy BE practices among all professionals.

**Conclusion:**

Though motivators vary among different sector professionals, opportunities may be the most important driver of healthy BE practices and potentially a target to improve multi-sector professional practices in Canada. Future research should confirm findings of this first study of professional practice drivers guided by a theoretical behaviour change framework.

**Supplementary Information:**

The online version contains supplementary material available at 10.17269/s41997-023-00824-y.

## Résumé

**Objectif:**

Avec la hausse des coûts des soins de santé liés aux maladies chroniques au Canada, les interventions visant à changer les comportements individuels en conditions cliniques doivent être complétées par une approche axée sur les déterminants de la santé, où des professionnels de plusieurs secteurs aident à créer des cadres communautaires plus sains. Notre étude vise à approfondir la compréhension des capacités, occasions, motivations et comportements (COM-B) des professionnels canadiens de plusieurs secteurs qui les amènent à travailler ensemble à améliorer les cadres bâtis favorisant la santé.

**Méthode:**

Nous avons mené une étude transversale auprès de 61 professionnels de plusieurs secteurs. Nous leur avons administré un sondage de 49 questions pour mesurer, selon les concepts du modèle COM-B, les pratiques favorisant des cadres bâtis sains.

**Résultats:**

Les professionnels de la santé publique (SP) étaient plus motivés par leurs valeurs/leur intérêt personnel envers les cadres bâtis sains et par l’existence de preuves scientifiques des effets de la conception des cadres bâtis sur la santé que les professionnels de la planification et les professionnels de l’élaboration de politiques/programmes (EPP). Les professionnels de la planification étaient plus susceptibles d’être motivés par les lois, les règlements et les codes sur les cadres bâtis sains que les professionnels de l’EPP. La pratique d’assumer la responsabilité de l’inclusion de caractéristiques saines dans la conception des cadres bâtis a été plus souvent déclarée par les professionnels de la planification et de l’EPP que par les professionnels de la SP. Pour l’ensemble des professionnels, les résultats affichent une tendance presque significative : les occasions pourraient être une variable prédictive de l’utilisation de pratiques saines en matière de cadre bâti.

**Conclusion:**

Les motivations des professionnels varient selon le secteur, mais les occasions pourraient être le principal moteur d’utilisation de pratiques saines en matière de cadres bâtis, et elles pourraient éventuellement être ciblées pour améliorer les pratiques professionnelles dans plusieurs secteurs au Canada. Les études futures devraient vérifier les constats de cette première étude sur les moteurs des pratiques professionnelles, guidée par un cadre théorique du changement des comportements.

## Introduction

The built environment (BE) represents an area for potential improvement that brings different stakeholders together for the common purpose of health. BE refers to human-made spaces where we live, work, and recreate. These include buildings; areas outside of buildings such as landscaping, streets, and neighbourhoods; and physical amenities available like parks, green spaces, sidewalks, bike paths, and transit networks (Leyden, 2003; National Research Council et al., 2005; Roof & Oleru, 2008; Sallis et al., 2012). History has shown the importance of environmental controls for infectious diseases transcending individual efforts (Centers for Disease Control & Prevention, 2019). Food and water safety, and ventilation in buildings are among the generally accepted and even expected measures today. The focus on BE improvements, rather than individualized efforts alone, may present an opportunity for improving critical health outcomes, including the huge burden of non-communicable diseases.

Research reveals that poorly designed BE (e.g. poorly located stairwells, non-existent or poorly maintained sidewalks, bike paths, transit networks, and green spaces) is associated with increased risks of chronic conditions and risk factors like physical inactivity, unhealthy diet, and social isolation (Codinhoto et al., 2009; Dixon et al., 2021; Nathan et al., 2018; Pinchoff et al., 2020; Prince et al., 2022; Rao et al., 2007; Renalds et al., 2010). With rapidly rising healthcare costs from chronic conditions (Elmslie, 2012; Mirolla, 2004; Public Health Agency of Canada, 2011; Tsasis & Bains, 2008), and such conditions serving as key risk factors for severe infection in pandemics like COVID-19 (Földi et al., 2020; Soeroto et al., 2020; Yang et al., 2021), increasing active living, healthy eating, and social connections are more critical than ever. Individual behaviour change interventions are limited, requiring environmental support (Cole et al., 2019; The Community Guide, 2022; Truman et al., 2000). In 2017, the Chief Public Health Officer of Canada issued a Call to Action for concerted efforts to improve BE to support healthy living (Public Health Agency of Canada, 2017).

Collaborations among different professionals from multiple sectors (hereafter called “multi-sector professionals”) are key to creating healthier BE. Work in non-Canadian jurisdictions taking comprehensive environmental and policy approaches shows success in improving chronic diseases and risk factors (Bartley et al., 2019; Day et al., 2020; Lee, 2020; Robbins et al., 2015). Likewise, these jurisdictions have documented the importance of multi-sector professional collaborations in improving BE for health (Kelly et al., 2016; Lee, 2012; Rube et al., 2014). Expanding on these initial studies with Canadian multi-sector professionals would support the understanding of healthy BE practices of multi-sector professionals and the potential for increasing collaborations in Canada. BE practices are nuanced; navigating laws and priorities of multiple stakeholder groups working on BE determines decision-making (Perdue et al., 2003). Studying factors shaping BE practices is important to ensure effective healthy BE evidence is translated into practice.

Research thus far has primarily focused on understanding professionals’ barriers to implementing healthy BE practices including inadequate funding and staffing, conflicting goals across different levels of government, lack of collaboration among professionals, lack of knowledge about health impacts of BE, inability to sustain improved BE design (e.g. sidewalk quality) over the long term, and costs (Bocarro et al., 2009; Carmichael et al., 2012; Dill & Howe, 2011; Evenson et al., 2011; Goins et al., 2013; Hollander et al., 2008). One qualitative study attempted to understand professional practice drivers (Pineo & Moore, 2021). A key limitation of studies has been the lack of guiding theoretical frameworks to ensure study comprehensiveness (Michie et al., 2011). Additionally, no published studies are from Canada.

Use of psychological models is recommended for understanding and changing behaviours (Michie et al., 2005, 2011). The capability, opportunity, motivation, and behaviour (COM-B) model (Atkins et al., 2017; Michie et al., 2011, 2014) combines existing health behaviour theories and comprises three main interacting components (capability, opportunity, and motivation) that influence specific behaviours (hereafter referred to as “practices”). These factors are used to identify the most effective interventions for behaviour and policy change (Atkins et al., 2017; Michie et al., 2014). Capability refers to physical and psychological competencies of individuals to engage in specific practices. Opportunity refers to external factors influencing specific practices, including physical and social factors. Motivation refers to brain processes informing specific practices, including reflective and automatic processes (Michie et al., 2011, 2014).

Though COM-B has been identified as a useful model for implementation science (Handley et al., 2016), and used in implementing evidence-based practices in healthcare and equitable dissemination efforts (Alexander et al., 2014; Baumann et al., 2022; De Leo et al., 2021), it has not been applied to implementation of healthy BE. We aim to gain insights into underlying COM-B factors enabling integration of physical activity (active transportation, active recreation, and active mobility in buildings), healthy food and beverage access, and social connections (hereafter called “healthy living”) into decision-making about BE. Through this process, we identify potential behavioural interventions to increase the implementation of healthy BE practices among multi-sector professionals. Our study attempts to answer these research questions:

1. What are capabilities, opportunities, and motivations of different professionals for implementing healthy BE practices?
2. What healthy BE practices do different professionals currently implement?
3. Is there a relationship between capability, opportunity, and motivation, and healthy BE practices among different professionals?

## Methods

### Study population

A cross-sectional case study occurred among professionals working on or interested in BE in Canada through participation in Housing for Health (H4H) initiatives including the Partnership Working Group (PWG) and annual Fit Cities Fit Towns (FCFT) Canada Conference. H4H is funded by the Public Health Agency of Canada to create healthier communities through multi-sector partnerships (www.uab.ca/h4h).

### Survey development

Using the COM-B framework, literature reviews of professionals’ experiences in implementing healthy BE practices (Bocarro et al., 2009; Carmichael et al., 2012, 2019; Dill & Howe, 2011; Evenson et al., 2011; Goins et al., 2013; Hollander et al., 2008; Lowe et al., 2018; Pineo & Moore, 2021; Pineo et al., 2020; Salvesen et al., 2008; Urban Land Institute, 2015; World Health Organization, 2020, 2022) were conducted. Questions were developed/adapted to measure constructs of COM-B for healthy BE practices. Survey items agreed upon by two or more H4H researchers were retained. Draft questions were then evaluated by other H4H team members for face validity (Miller & Lovler, 2018). Based on feedback, the term “BE design” was reworded to “community and/or building (CB)-design” for clarity. Additionally, “physical activity, healthy food and beverage access, and social connections” were referred to as “healthy living”. Reliability of the COM-B outcomes was assessed via Cronbach’s alpha values and were between 0.73 and 0.93, indicating acceptable reliability (Taber, 2018). The final survey evaluated 49 COM-B and six demographic items.

Capability was measured on a 7-item scale assessing professionals’ knowledge of health impacts of CB-design. Participants indicated whether different health outcomes are influenced by CB-design with responses: No (0), Don’t know (1), Maybe (2), and Yes (3). Items were summed with total scores up to 21 points.

Opportunity was measured on an 18-item scale assessing professionals’ access to resources and perceived supports enabling healthy living integration into CB-design. Likewise, motivators/drivers of considering healthy living in CB-design decision-making were assessed on an 18-item scale. These items were rated on a 5-point Likert scale ranging from strongly disagree (1) to strongly agree (5) and summed for total scores of 18–90 points.

Healthy BE practices were measured by asking professionals to rate how frequently they engaged in six actions in healthy living integration into CB-design decision making. Items on a 5-point Likert scale ranged from never (1) to always (5) and summed for total scores of 6–30 points.

Job function had 18 items, categorized into 3 main job functions including public health (PH), planning, and those working on policy and/or developing/implementing/maintaining programs, hereafter called “policy/program development (PPD) professionals”. Due to small samples of other professionals, a fourth group “other professionals” was created.

### Survey administration

Personalized emails with survey link were sent to registrants of the 2022 FCFT virtual conference in February 2022 and to members of H4H PWG in February 2022 and 2023. The 2023 survey included a question about previous participation in the 2022 survey.

### Ethics approval

This study was approved by the University of Alberta Research Ethics Board.

### Data analysis

Data were analyzed using SAS software version 9.4. Descriptive statistics including medians, counts, and percentages were computed for constructs of COM-B for the full sample and stratified by job function. ANOVA and Kruskal–Wallis tests were conducted to examine differences in variables across different BE professionals. The Dwass-Steel-Critchlow-Fligner (DSCF) multiple comparisons post hoc test was conducted to determine which pairs of professionals differ. To examine relationships between COM constructs and healthy BE practices, univariate linear regressions were first conducted to examine individual relationships of each COM construct and healthy BE practices for all professionals and stratified by job function. Then, multivariate linear regressions were conducted to examine similar relationships for all professionals and stratified by job function. A multivariate regression analysis with three exposure variables requires 76 participants to detect medium size effects with 80% power and *p* < 0.05 (Cohen, 2016). Effect sizes (adjusted *R*^2^ values) were interpreted as 0.02 = small effect, 0.15 = medium effect, and 0.35 = large effect (Cohen, 2016), and were used with significance values to interpret results. Statistical values were considered significant at *p* < 0.05. Trends towards significance (*p* < 0.10) were also noted.

## Results

In 2022, surveys were sent to 202 H4H PWG members and 59 FCFT Conference registrants, of whom 74 completed the survey (28% response rate). In 2023, surveys were sent to 244 H4H PWG members, of whom 37 completed the survey (15% response rate). Twenty-four of the 2023 survey participants completed the 2022 survey and were excluded. Focusing on currently practicing professionals, surveys by students, postdoctoral fellows, retired professionals, and those who did not provide information about their current job functions were also excluded, leaving a final sample of 61. Table 1 shows most participants were ≥ 35 years old (75%), female (65%), white (84%), and with university degrees (90%). Most had been in their profession for ≥ 5 years (70%).

**Table 1:** Demographic characteristics of study participants (*N* = 61)

Demographics	*n*(%)
Age	
Under 25 years	0
25–34 years	15(24.6)
35–44 years	21(34.4)
45–54 years	12(19.7)
55 years or older	13(21.3)
Gender	
Male	18(30)
Female	39(65)
Other	3(5)
Race/ethnicity	
White	48(84.2)
Non-white	9(15.8)
Education	
Secondary (high) school or less	0
Trades certificate or non-university certificate	4(8.2)
University certificate or diploma below bachelor level	1(1.6)
Bachelor’s degree	19(31.2)
University certificate or diploma above bachelor level	3(4.9)
Degree in medicine, dentistry, veterinary medicine, or optometry	5(8.2)
Master’s degree or higher	28(45.9)
Length of work	
Under 5 years	18(30)
5 to 10 years	15(25.0)
11 to 15 years	3(5.0)
16 to 20 years	10(16.7)
Over 20 years	14(23.3)
Job function	
Public health professional	12(19.7)
Planning professional	15(24.6)
Policy/Program development professional^a^	14(23)
Other^b^	20(32.8)

^a^‘Policy/program development professional’ comprises professionals who work on policy issues and/or develop/implement/maintain programs. ^b^‘Other’ comprises healthcare professionals, academics/researchers, architects, landscape architects/professionals, urban design professionals, interior design professionals, engineering professionals, retail, market housing, or affordable housing developer

### Capability

High percentages (> 90%) of participants correctly identified CB-design influencing physical activity, safety, social connections, healthy eating, access to food, and health outcomes. When stratified by job function, > 90% of PH professionals, > 79% of planning professionals, > 90% of PPD professionals, and > 89% of other professionals identified CB-design influencing relevant health outcomes (Table S1). A Kruskal–Wallis test showed no statistically significant differences in capability between different professionals (*p* = 0.2033) (Table 2).

**Table 2:** Differences in the capability (i.e., knowledge of factors such as physical activity, safety outcomes, social connections, healthy eating, access to food, and health outcomes influenced by community and/or building design) among different built environment professionals

	Public health professionals Median	Planning professionals Median	Policy/program development professionals Median	Other professionals^a^ Median	Kruskal–Wallis test *p*-value
Sum of factors	21	21	21	20.5	0.2033

^a^‘Other’ comprises healthcare professionals, academic/researchers, architects, landscape architects/professionals, urban design professionals, interior design professionals, engineering professionals, retail, market housing, or affordable housing developer

### Opportunity

The most reported opportunities enabling healthy living integration into CB-design decision-making were presence of informal networks (61%) and scientific evidence (56%). Least were presence of taxation/subsidies for healthy CB (49%), government/political support (49%), and human resources (43%).

Stratified, the most reported opportunity among PH professionals was available scientific evidence of CB-design impacts on healthy living (83%); among planning professionals, presence of professional associations (60%); and among PPD (71%) and other professionals (60%), presence of informal networks. Least reported opportunities by job function were taxation/subsidies for healthy CB by PH (58%) and planning professionals (60%), and government/political support by PPD (57%) and other professionals (60%) (Table S2).

Separate Kruskal–Wallis tests for each individual opportunity item showed no differences in scores across different professionals (all *p*-values > 0.081; Table S2). Likewise, there was no statistically significant difference in summary scores (*p*-value = 0.997; data not shown).

### Motivation

Among all professionals, the most rated motivator for integrating healthy living into CB-designs was interest in societal/community impacts of CB-designs (98%); least was healthy BE legislation/regulations/codes (30%).

Stratified, the most reported motivator was interest in societal/community impacts of CB-designs among PH (100%), planning (100%), PPD (100%), and other professionals (95%). Healthy BE legislation/regulations/codes were least reported among PH (33%), PPD (43%), and other professionals (35%). Among planning professionals, mindfulness of assets and reputation were the least reported motivator (27%) while healthy BE legislation/regulations/codes were the most reported motivators (80%; Table S3).

Kruskal–Wallis tests showed no difference in motivation summary scores among different professionals (*p* = 0.286; data not shown); however, for individual motivation items, four motivators were statistically different across professional groups including presence of healthy BE legislation/regulations/codes (*p* = 0.013), personal interest/values in healthy CB-designs (*p* = 0.007), and scientific evidence on CB-design health impacts (*p* = 0.027) (Table S3). DSCF tests showed a statistically significant difference for personal interest/values in healthy CB-designs with PH more likely to *strongly agree* (median = 5) with the motivator compared to planning professionals (median = 4; *p* = 0.049) or PPD professionals (median = 4; *p* = 0.022)*.* Likewise, there was a statistically significant difference for presence of healthy BE legislation/regulations/codes with planning professionals more likely to *agree* (median = 4) with the motivator compared to PPD professionals (median = 2; *p* = 0.017). Finally, there was a statistically significant difference for presence of scientific evidence on CB-design health impacts with PH more likely to *strongly agree* (median = 5) with the motivator compared to planning professionals (median = 4; *p* = 0.045).

### Healthy BE practices

The most reported healthy BE practice among all professionals was collaborating and sharing knowledge with partners around implementing healthy CB-design features (54%). Least was evaluating impacts of healthy CB-implementation (48%) (Table S4).

Stratified, collaborating and sharing knowledge with partners was the most reported among PH (42%), PPD (64%), and other professionals (63%). Advocating for inclusion of healthy features into CB-design was the most reported among planning professionals (57%). Least reported was evaluating impacts of healthy CB implementation for PH (83%), planning (50%), and other professionals (42%). Providing scientific evidence on healthy living impacts of CB-designs to stakeholders was the least reported practice for PPD professionals (36%). Although professionals reported different practices, taking responsibility for healthy feature inclusion into CB-design (*p* = 0.007) and evaluating the degree of healthy CB implementation (*p* = 0.047) were statistically significantly different (Table S4). DSCF tests showed a statistically significant difference for taking responsibility for healthy feature inclusion in CB-design with PH (median = 2) less likely to *agree* with this practice compared to planning (median = 3; *p* = 0.015) and other professionals (median = 3.5; *p* = 0.013).

### Relationship between COM constructs and healthy BE practices

In univariate linear regressions for all professionals, opportunities accounted for 6.2% of variance in practices, and there was a trend towards significance (*β* = 0.11, SE = 0.06, *p* = 0.062; Table 3). Stratified by job function, there was a statistically significant association between opportunities and healthy BE practices among PH professionals (*β* = 0.24, *p* = 0.049) with 33.2% of variance in practices explained (Table 3). For other job functions, no significant associations were observed.

**Table 3:** Univariate linear relationship between COM constructs and healthy built environment practices^a^

	Healthy built environment practices				
COM constructs	*β* estimate (standard error (SE), *p*-value, *R* squared values)				
	All professionals	Public health professionals	Planning professionals	Policy/program development professionals	Other professionals^b^
Capability	0.08 (*SE* = *0.31, p* = *0.7879, R*^*2*^ = *0.001*)	− 1.01 (*SE* = *1.23,* *p* = *0.434, R*^*2*^ = *0.062*)	0.03 (*SE* = *0.37,* *p* = *0.9395, R*^*2*^ = *0.001*)	0.32 (*SE* = *0.79,* *p* = *0.6956, R*^*2*^ = *0.015*)	0.79 (*SE* = *0.80,* *p* = *0.3348, R*^*2*^ = *0.058*)
Opportunities	0.11 (*SE* = *0.06,* *p* = *0.0620, R*^*2*^ = *0.062*)	0.24 (*SE* = *0.11; p* = 0.049**; R^*2*^ = *0.332*)	0.14 (*SE* = *0.07, p* = *0.0758, R*^*2*^ = *0.239*)	0.18 (*SE* = *0.16,* *p* = *0.3049, R*^*2*^ = *0.095*)	− 0.01 (*SE* = *0.11,* *p* = *0.8914, R*^*2*^ = *0.001*)
Motivation	0.04 (*SE* = *0.06,* *p* = *0.5362, R*^*2*^ = *0.007*)	0.01 (*SE* = *0.07, p* = *0.9204, R*^*2*^ = *0.001*)	0.20 (*SE* = *0.12,* *p* = *0.1103, R*^*2*^ = *0.199*)	0.21 (*SE* = *0.16,* *p* = *0.2064, R*^*2*^ = *0.141*)	− 0.24 (*SE* = *0.17,* *p* = *0.1697, R*^*2*^ = *0.114*)

^a^The model contained only one independent variable, i.e. one of the constructs (e.g. capability)

^b^‘Other’ comprises healthcare professionals, academic/researchers, architects, landscape architects/professionals, urban design professionals, interior design professionals, engineering professionals, retail, market housing, or affordable housing developer

^**^p < 0.05

In multivariate linear regression for all professionals, no statistically significant associations occurred between COM and healthy BE practices (0.9% of variance in practices explained). There was a trend towards significance for opportunities (*β* = 0.11, SE = 0.06, *p* = 0.088). Stratified by job function, no significant associations were observed (Table 4). However, a trend towards significance between opportunities and increased healthy BE practices was observed among PH professionals (*β* = 0.25, SE = 0.12, *p* = 0.063) with 18.5% of variance in practices explained (Table 4).

**Table 4:** Multivariate linear relationship between capability, opportunity, and motivation (COM) constructs and healthy built environment practices^a^

	Healthy built environment practices				
COM constructs	*β* estimate (standard error, *p-*value)				
	All professionals	Public health professionals	Planning professionals	Policy/program development professionals	Other professionals^b^
Capability	0.01 (*SE* = *0.31* *,p* = *0.9787*)	− 1.10 *(SE* = *1.10,* *p* = *0.347)*	− 0.25 (*SE* = *0.38,* *p* = *0.5211*)	0.42 (*SE* = *0.81,* *p* = *0.6128*)	0.26 (*SE* = *0.93;* *p* = *0.7799*)
Opportunities	0.11 (*SE* = *0.06,* *p* = *0.0878*)	0.25 (*SE* = *0.12, p* = *0.063*)	0.13 (*SE* = *0.10,* *p* = *0.2300*)	0.04 (*SE* = *0.24,* *p* = *0.8708*)	0.09 (*SE* = *0.13;* *p* = *0.4859*)
Motivation	− 0.01 (*SE* = *0.07,* *p* = *0.8765*)	0.003 (*SE* = *0.06, p* = *0.957*)	0.10 (*SE* = *0.15,* *p* = *0.5111*)	0.19 (*SE* = *0.25, p* = *0.4549*)	− 0.30 (*SE* = *0.24,* *p* = *0.2296*)
Overall adjusted *R*^2^	0.009	0.185	0.106	− 0.107	− 0.028

^a^The model contained all the constructs (capability, opportunity, and motivation) as independent variables

^b^‘Other’ comprises healthcare professionals, academic/researchers, architects, landscape architects/professionals, urban design professionals, interior design professionals, engineering professionals, retail, market housing, or affordable housing developer

## Discussion

Studies from different non-Canadian jurisdictions have noted the importance of multi-sector professional practices in improving our BE for health (Kelly et al., 2016; Lee, 2012; Rube et al., 2014). Barriers to improving multi-sector professionals’ practices have been studied. However, no such published studies are from Canada, and available studies do not use a theoretical framework to guide inquiry. Guided by the COM-B model (Michie et al., 2011), this study aimed to understand comprehensive drivers of practices among Canadian multi-sector professionals, who must work together for healthy BE implementation.

Professionals working on BE are generally knowledgeable about health impacts of BE, with no significant differences across job functions. This finding is not surprising considering current extensive literature on BE and health (Codinhoto et al., 2009; Rao et al., 2007; Renalds et al., 2010), and increased interests in this topic in practice in recent years (Jackson et al., 2013). This finding may also be due to study recruitment of participants involved in healthy BE initiatives. Consistent with our findings, a qualitative study reported adequate knowledge about health impacts of BE among different professionals (Pineo & Moore, 2021). Along with lack of association with healthy BE practices, findings suggest interventions focused solely on increasing knowledge may not be effective at increasing healthy BE practices across multi-sector professionals. Future research should confirm these results.

Presence of informal networks, scientific evidence of CB-design impacting healthy living, and professional associations were the most reported opportunities enabling healthy BE practices among different professionals. Findings are consistent with a qualitative study where professional bodies and informal networks were essential factors for sharing BE implementation practices and capacity-building (Pineo & Moore, 2021). Other studies have highlighted case studies, guidelines, frameworks, standards, inter-sectoral collaborations, and knowledge-sharing among formal and informal networks as supporting healthy BE implementation (Carmichael et al., 2012, 2019; Lowe et al., 2018; Pineo et al., 2020; Urban Land Institute, 2015; World Health Organization, 2020, 2022).

Least reported opportunities for healthy BE implementation across all professionals included available taxation/subsidies for healthy CB; government/political support; legislation/regulations/codes; non-government or private-sector support; healthy CB certification systems/criteria; supportive organizational structure and culture; and human resources. Previous studies (Bocarro et al., 2009; Dill & Howe, 2011; Evenson et al., 2011; Goins et al., 2013; Hollander et al., 2008; Salvesen et al., 2008) highlighted barriers to healthy BE practices as including lack of such opportunities. Considering small-to-medium effect sizes and the trend towards significance for opportunities predicting healthy BE practices for all professionals, improving opportunities may be a key target for interventions in Canada, especially for PH professionals (medium-to-large effect size). Future research is needed to further test this hypothesis.

Integrating healthy living into CB-designs, interest in societal/community impacts of CB-designs and personal interest/values in healthy CB-designs were the most reported motivators among all professionals, consistent with motivators of professionals globally (Pineo & Moore, 2021). We also found PH professionals more motivated by personal interest/values and available scientific evidence for CB-design health impacts compared to planning and PPD professionals. Planning professionals were more motivated by healthy BE legislation/regulations/codes compared to PPD professionals. However, overall, motivations were not associated with healthy BE practices. With non-meaningful effect sizes (~ zero), interventions may not want to target only motivations. Future research should confirm this.

Among all professionals working on BE, collaborating and sharing knowledge with partners around implementing healthy-CB-design features was a top practice. This finding is supported by Pineo and Moore’s research (Pineo & Moore, 2021) but conflicts with others (Dill & Howe, 2011; Goins et al., 2013; Salvesen et al., 2008). Differences in findings could be due to our study sample of professionals involved with healthy BE projects. We also found planning and other professionals more likely to report taking responsibility for the inclusion of healthy features into CB-design compared to PH professionals, potentially reflecting the direct control planning and other professionals may have over BE matters (e.g. planning neighbourhoods/developments) while PH professionals may only advise or advocate on BE designs.

Our findings have implications for interventions promoting healthy BE practices among different professionals working together on BE. Interventions could target increasing opportunities for all professionals to integrate healthy living into CB-designs. Lack of current government and non-government support cited for integrating healthy living in CB-design is a potential opportunity. Government support may include improving public policies. Such policies may in turn be supported by sharing scientific evidence of health, environment, and economic impacts of improved BE with health professionals involved in policymaking. Both government and non-government support could also be increased through demonstrating community support for healthy BE, and feasibility and tangible successes of healthy BE implementation case studies (Brownson et al., 2009; Carlson et al., 2011). Evaluations of information-sharing forums like FCFT conferences have shown they help increase COM for healthy BE practices. To address lack of supportive organizational structure and culture, and human resources, different sectors could prioritize healthy BE work, and structure organization management and activities to support it. Studies have reported that leaders of public agencies who focused their departments on healthy BE work achieved significant BE changes (Kelly et al., 2016; Kuiper et al., 2012; Lee, 2012, 2020; Rube et al., 2014). Incentives like taxation/subsidies for healthy CB could be encouraged (NYC Economic Development Corporation, 2009); Rydin, 2012). In the United States, based on multi-sector professional feedback, Fannie Mae created the Healthy Housing Rewards initiative. City of New York created the Food Retail Expansion to Support Health program, providing financial and zoning incentives for healthy BE features (Fannie Mae, 2023; NYC Economic Development Corporation, 2009). Similar incentives could potentially improve healthy BE practices in Canada. An additional opportunity could be to increase routine use of healthy buildings and communities-related certification systems, and available guidelines, such as the Healthy Community Guidelines co-developed by over 100 multi-sector professionals in multiple Canadian provinces (Enterprise Community Partners, 2004; Housing for Health, 2023; International WELL Building Institute, 2018; McArthur & Powell, 2020; Pineo & Rydin, 2018; U.S. Green Building Council, 2009).

To our knowledge, this is the first study to explore healthy BE practices among different professionals based on a theoretical framework. Using the COM-B model allowed identification of comprehensive drivers of professional practices, including both intrinsic and extrinsic factors, that identify potential interventions among multi-sector professionals. Other frameworks related to policy change may also be further explored in future studies to identify additional extrinsic factors (Moloughney, 2012).

This study has several limitations. First, findings are from a convenience sample of Canadian professionals working on BE and involved in H4H initiatives. This sample may not capture the COM-B perspectives of the full range of sectors working on BE in Canada, nor be a representative example of the sectors captured in the study. Findings, therefore, may not generalize to all professionals working on BE. Second, although 87 survey respondents were greater than sample size required to power statistical calculations, currently non-practicing professionals were excluded, leaving a sample of 61 respondents for analyses. Insufficient power may have limited our ability to detect additional statistically significant associations and draw more definitive conclusions. Third, the category of “other professionals” includes very diverse job functions ranging from designers to developers to engineers. Fourth, high percentages of study participants were female and white. Although previous studies have shown females and white populations are more likely to participate in research (Glass et al., 2015; Webb et al., 2019), the higher percentage of females and whites in our study may not reflect distributions of sex/gender and race among BE professionals. Since a literature search did not find available studies showing sex/gender distributions for BE disciplines, future research could work with various professional associations to determine such distributions in different professions. Fifth, our survey was administered at two time periods to increase sample size. However, an independent *t*-test found no differences in COM-B between 2022 and 2023 survey respondents. Future research should recruit larger, more representative, and diverse samples of professionals working on BE with sufficient participants in each job function and sociodemographic category. Finally, since this was a cross-sectional study, causal interpretations of associations cannot be made.

## Conclusion

Evidence has been growing for improving BE as a key way to improve public health. To improve BE, participation of multi-sector professionals is essential. Identifying and understanding comprehensive practice drivers of different professional groups is therefore critical. This study highlights that, though motivators vary among different sector professionals in Canada, opportunities may be the most important driver of healthy BE practices, and interventions increasing opportunities may be most effective at producing change. As the first study guided by a theoretical behaviour change framework, future research should confirm findings.

## Contributions to knowledge

What does this study add to existing knowledge?

- Successful implementation of healthy BE for supporting health and well-being of our populations, and healthcare system sustainability requires multi-sector professionals working to improve our environments.
- Studies from different non-Canadian jurisdictions taking comprehensive community environmental and policy approaches have shown successes in improving chronic disease and risk factor outcomes. Expanding on these initial studies with Canadian multi-sector professionals would support understanding and improving healthy BE practices of multi-sector professionals in Canada.
- Barriers to improving multi-sector professionals’ practices have been studied. However, no such studies used a theoretical framework to guide inquiry or were conducted in Canada. This study used the COM-B model of behaviour change theoretical framework to guide assessment of the comprehensive drivers of professional practices.

What are the key implications for public health interventions, practice, or policy?

- To promote healthy BE practices among different Canadian professionals working on BE, increasing opportunities for all professionals to integrate healthy living into CB-designs may be the most effective approach.
- Lack of current government and non-government supports cited for integration of healthy living in CB-design are potential opportunities. Other opportunities include sharing scientific evidence of health impacts of improved BE, especially with PH professionals; prioritizing healthy BE work; and structuring organization management and activities for healthy CB-designs.
- Incentives and routine use of healthy buildings and communities-related certification systems and guidelines could be encouraged.

### Supplementary Information

Below is the link to the electronic supplementary material.

Supplementary file1 (DOCX 83 KB)

## Data Availability

Not applicable.
