# Supplementary material for: Capabilities, opportunities, motivations, and practices of different sector professionals working on community environments to improve health
Source: Can J Public Health. 2023 Nov 2;115(1):132–42. doi: 10.17269/s41997-023-00824-y (PMC10853135; doi:10.17269/s41997-023-00824-y)
Supplement: Supplementary file 1 — Supplementary file1 (DOCX 83 KB) [file 41997_2023_824_MOESM1_ESM.docx]

**Supplementary File**

Table S1: Summary of capability scores (i.e., knowledge of factors such as physical activity, safety outcomes, social connections, healthy eating, access to food, health outcomes influenced by community and/or building design), stratified by job function.

| **Survey Question:** *Which of the following do you think is influenced by community and/or building design?* | *Response scales^a^* | **All professionals**  n (%) | **Public Health professionals**  n (%) | **Planning professionals**  n (%) | **Policy/program development professionals**  n (%) | **Other professionals**  n (%) |
| --- | --- | --- | --- | --- | --- | --- |
| 1. *Physical activity levels of residents* | *Yes* | 58(96.7) | 12(100) | 14(93.3) | 13(92.9) | 19(100) |
|  | *No* | 2(3.3) | 0 | 1(6.7) | 1(7.1) | 0 |
|  | *Don’t know* | 0 | 0 | 0 | 0 | 0 |
| 1. *Health outcomes of residents such as obesity, heart disease, diabetes, and cancers* | *Yes* | 56(91.8) | 12(100) | 13(86.7) | 13(92.9) | 18(90) |
|  | *No* | 5(8.2) | 0 | 2(13.3) | 1(7.1) | 2(10) |
|  | *Don’t know* | 0 | 0 | 0 | 0 | 0 |
| 1. *Access to or eating of healthy or unhealthy foods and beverages* | *Yes* | 56(91.8) | 12(100) | 12(80) | 13(92.9) | 19(95.0) |
|  | *No* | 5(8.2) | 0 | 3(20) | 1(7.1) | 1(5.0) |
|  | *Don’t know* | 0 | 0 | 0 | 0 | 0 |
| 1. *Safety from crime* | *Yes* | 58(95.1) | 12(100) | 13(86.7) | 14(100) | 19(95.0) |
|  | *No* | 3(4.92) | 0 | 2(13.3) | 0 | 1(5.0) |
|  | *Don’t know* | 0 | 0 | 0 | 0 | 0 |
| 1. *Safety from traffic* | *Yes* | 60(98.4) | 12(100) | 14(93.3) | 14(100) | 20(100) |
|  | *No* | 1(1.6) | 0 | 1(6.7) | 0 | 0 |
|  | *Don’t know* | 0 | 0 | 0 | 0 | 0 |
| 1. *Social connections of residents* | *Yes* | 61(100) | 12(100) | 15(100) | 14(100) | 20(100) |
|  | *No* | 0 | 0 | 0 | 0 | 0 |
|  | *Don’t know* | 0 | 0 | 0 | 0 | 0 |
| 1. *Climate change* | *Yes* | 55(91.7) | 11(91.6) | 13(86.7) | 14(100) | 17(89.5) |
|  | *No* | 5(8.3) | 1(8.3) | 2(13.3) | 0 | 2(10.5) |
|  | *Don’t know* | 0 | 0 | 0 | 0 | 0 |

^a^Responses of “yes” and “maybe” are summed and displayed under the option “yes”.

Table S2: Summary of opportunity item scores that enable integration of health in decision-making about community design, stratified by job function.

| **Survey Question:**  *To what extent do you agree that the following are available in your position to enable decision-making about community and/or building design for healthy living?* | *Response scales^a^* | **All professionals**  n (%) | **Public Health professionals**  n (%) | **Planning professionals**  n (%) | **Policy/program development professionals**  n (%) | **Other professionals**  n (%) | *Kruskal-Wallis Test*  *p-value* |
| --- | --- | --- | --- | --- | --- | --- | --- |
| 1. *Healthy community and/or building legislation, regulations, and codes* | *Agree* | 20(32.8) | 3(25.0) | 6(40) | 3(21.4) | 8(40) | *0.5381* |
|  | *Neutral* | 14(23.0) | 2(16.7) | 2(13.3) | 4(28.6) | 6(30) |  |
|  | *Disagree* | 20(32.8) | 6(50.0) | 5(33.3) | 5(35.7) | 4(20) |  |
|  | *Don’t know* | 5(8.2) | 1(8.3) | 1(6.7) | 2(14.3) | 1(5) |  |
|  | *N/A* | 2(3.3) | 0 | 1(6.7) | 0 | 1(5) |  |
|  | **Median**^b^ |  | 2 | 3 | 3 | 3 |  |
| 1. *Healthy community and/or building standards* | *Agree* | 18(29.5) | 3(25.0) | 7(46.7) | 3(21.4) | 5(25) | *0.8029* |
|  | *Neutral* | 19(31.2) | 5(41.7) | 3(20.0) | 4(28.6) | 7(35) |  |
|  | *Disagree* | 17(27.9) | 3(25.0) | 4(26.7) | 4(28.6) | 6(30) |  |
|  | *Don’t know* | 6(9.8) | 1(8.3) | 1(6.7) | 3(21.4) | 1(5) |  |
|  | *N/A* | 1(1.6) | 0 | 0 | 0 | 1(5) |  |
|  | **Median**^b^ |  | 3 | 3.5 | 3 | 3 |  |
| 1. *Healthy community and/or building guidelines* | *Agree* | 31(50.8) | 7(58.3) | 8(53.3) | 7(50) | 9(45) | *0.8259* |
|  | *Neutral* | 11(18.0) | 2(16.7) | 3(20.0) | 2(14.3) | 4(20) |  |
|  | *Disagree* | 14(23.0) | 2(16.6) | 3(20) | 4(28.6) | 5(25) |  |
|  | *Don’t know* | 4(6.6) | 1(8.3) | 1(6.7) | 1(7.1) | 1(5) |  |
|  | *N/A* | 1(1.6) | 0 | 0 | 0 | 1(5) |  |
|  | **Median**^b^ |  | 4 | 4 | 4 | 3.5 |  |
| 1. *Healthy community and/or building certification systems or criteria* | *Agree* | 18(29.5) | 0 | 3(20) | 6(28.6) | 9(45) | *0.0882* |
|  | *Neutral* | 11(18.0) | 4(33.3) | 2(13.3) | 2(14.3) | 3(15 |  |
|  | *Disagree* | 20(32.8) | 4(33.4) | 8(53.3) | 3(21.4) | 5(25) |  |
|  | *Don’t know* | 11(18.0) | 4(33.3) | 2(13.3) | 3(21.4) | 2(10) |  |
|  | *N/A* | 1(1.6) | 0 | 0 | 0 | 1(5) |  |
|  | **Median**^b^ |  | 2.5 | 2 | 4 | 4 |  |
| 1. *Scientific evidence of the impact of community and/or building design on healthy living* | *Agree* | 34(55.7) | 10(83.4) | 7(46.7) | 6(42.9) | 11(55) | *0.3823* |
|  | *Neutral* | 7(11.5) | 1(8.3) | 1(6.7) | 4(28.6) | 1(5) |  |
|  | *Disagree* | 13(21.3) | 1(8.3) | 5(33.3) | 2(14.3) | 5(25) |  |
|  | *Don’t know* | 5(8.2) | 0 | 1(6.7) | 2(14.3) | 2(10) |  |
|  | *N/A* | 2(3.3) | 0 | 1(6.7) | 0 | 1(5) |  |
|  | **Median**^b^ |  | 4 | 4 | 3.5 | 4 |  |
| 1. *Professional associations (e.g., Canadian Institute of Planners)* | *Agree* | 29(47.5) | 6(50.0) | 9(60) | 6(42.9) | 8(40) | *0.7628* |
|  | *Neutral* | 11(18.0) | 1(8.3) | 4(26.7) | 1(7.1) | 5(25) |  |
|  | *Disagree* | 11(18.0) | 3(25.0) | 2(13.3) | 3(21.4) | 3(15) |  |
|  | *Don’t know* | 9(14.8) | 2(16.7) | 0 | 4(28.6) | 3(15) |  |
|  | *N/A* | 1(1.6) | 0 | 0 | 0 | 1(5) |  |
|  | **Median**^b^ |  | 4 | 4 | 4 | 3.5 |  |
| 1. *Formal networks of people across work or volunteer departments/units/areas/agencies* | *Agree* | 28(39.9) | 8(66.7) | 6(40) | 5(35.7) | 9(45.0) | *0.9111* |
|  | *Neutral* | 14(23.0) | 0 | 6(40) | 4(28.6) | 4(20) |  |
|  | *Disagree* | 14(23.0) | 4(33.3) | 2(13.3) | 3(21.4) | 5(25) |  |
|  | *Don’t know* | 3(4.9) | 0 | 0 | 2(14.3) | 1(5) |  |
|  | *N/A* | 2(3.3) | 0 | 1(6.7) | 0 | 1(5) |  |
|  | **Median**^b^ |  | 4 | 3 | 3 | 3.5 |  |
| 1. *Informal networks of people* | *Agree* | 37(60.7) | 9(75.0) | 6(40) | 10(71.4) | 12(60) | *0.6220* |
|  | *Neutral* | 11(18.0) | 2(16.7) | 5(33.3) | 2(14.3) | 2(10) |  |
|  | *Disagree* | 7(11.5) | 1(8.3) | 2(13.3) | 1(7.1) | 3(25) |  |
|  | *Don’t know* | 5(8.2) | 0 | 2(13.3) | 1(7.1) | 2(10.0) |  |
|  | *N/A* | 1(1.6) | 0 | 0 | 0 | 1(5) |  |
|  | **Median**^b^ |  | 4 | 3 | 4 | 4 |  |
| 1. *Pilot or demonstration projects* | *Agree* | 26(42.6) | 5(41.7) | 5(33.3) | 7(50) | 9(45) | *0.6698* |
|  | *Neutral* | 8(13.1) | 2(16.7) | 1(6.7) | 2(14.3) | 3(15) |  |
|  | *Disagree* | 19(31.2) | 4(33.4) | 7(46.7) | 3(21.4) | 5(25) |  |
|  | *Don’t know* | 7(11.5) | 1(8.3) | 2(13.3) | 2(14.3) | 2(10) |  |
|  | *N/A* | 1(1.6) | 0 | 0 | 0 | 1(5) |  |
|  | **Median**^b^ |  | 3 | 2 | 4 | 4 |  |
| 1. *Taxation or subsidies for healthy communities and/or buildings* | *Agree* | 7(11.5) | 0 | 2(13.3) | 1(7.1) | 4(20) | *0.0833* |
|  | *Neutral* | 10(16.4) | 1(8.3) | 0 | 3(21.4) | 6(30) |  |
|  | *Disagree* | 30(49.2) | 7(58.3) | 9(60) | 6(42.9) | 8(40) |  |
|  | *Don’t know* | 12(19.7) | 3(25.0) | 4(26.7) | 4(28.6) | 1(5) |  |
|  | *N/A* | 2(3.3) | 1(8.3) | 0 | 0 | 1(5) |  |
|  | **Median**^b^ |  | 1.5 | 1 | 2 | 3 |  |
| 1. *Courses/training programs/conferences/workshops* | *Agree* | 27(44.3) | 8(66.7) | 6(40) | 5(35.7) | 8(40) | *0.2764* |
|  | *Neutral* | 8(13.1) | 1(8.3) | 2(13.3) | 2(14.3) | 3(15) |  |
|  | *Disagree* | 19(31.2) | 2(16.7) | 4(26.7) | 6(42.9) | 7(35) |  |
|  | *Don’t know* | 5(8.2) | 1(8.3) | 2(13.3) | 1(7.1) | 1(5) |  |
|  | *N/A* | 2(3.3) | 0 | 1(6.7) | 0 | 1(5) |  |
|  | **Median**^b^ |  | 4 | 3.5 | 3 | 3 |  |
| 1. *Funding or grants* | *Agree* | 20(32.8) | 5(41.7) | 2(13.3) | 5(35.7) | 8(40) | *0.2984* |
|  | *Neutral* | 14(23.0) | 3(25.0) | 4(26.7) | 3(21.4) | 4(20) |  |
|  | *Disagree* | 20(32.8) | 1(8.3) | 6(40) | 5(35.7) | 8(40) |  |
|  | *Don’t know* | 5(8.2) | 2(16.7) | 2(13.3) | 1(7.1) | 0 |  |
|  | *N/A* | 2(3.3) | 1(8.3) | 1(6.7) | 0 | 0 |  |
|  | **Median**^b^ |  | 4 | 2.5 | 3 | 3 |  |
| 1. *Community support* | *Agree* | 25(41) | 4(33.3) | 5(33.3) | 8(57.1) | 8(40) | *0.7123* |
|  | *Neutral* | 17(27.9) | 4(33.3) | 6(40) | 3(21.4) | 4(20) |  |
|  | *Disagree* | 15(24.6) | 2(16.6) | 3(20) | 2(14.3) | 8(40) |  |
|  | *Don’t know* | 3(4.9) | 2(16.7) | 0 | 1(7.1) | 0 |  |
|  | *N/A* | 1(1.6) | 0 | 1(6.7) | 0 | 0 |  |
|  | **Median**^b^ |  | 3 | 3 | 4 | 3 |  |
| 1. *Human resources (e.g., staff)* | *Agree* | 16(26.7) | 4(33.3) | 4(28.6) | 4(28.6) | 4(20) | *0.9451* |
|  | *Neutral* | 12(20) | 2(16.7) | 3(21.4) | 4(28.6) | 3(15) |  |
|  | *Disagree* | 26(43.3) | 6(50.0) | 5(35.7) | 5(35.7) | 10(50) |  |
|  | *Don’t know* | 3(5.0) | 0 | 0 | 1(7.1) | 2(10) |  |
|  | *N/A* | 3(5.0) | 0 | 2(14.3) | 0 | 1(5) |  |
|  | **Median**^b^ |  | 2.5 | 3 | 3 | 2 |  |
| 1. *Supportive organizational structure and culture* | *Agree* | 22(36.7) | 4(33.3) | 7(46.7) | 4(30.7) | 7(35) | *0.5250* |
|  | *Neutral* | 13(21.7) | 2(16.7) | 4(26.7) | 4(30.7) | 3(15) |  |
|  | *Disagree* | 21(35) | 5(41.7) | 2(13.3) | 4(30.7) | 10(50) |  |
|  | *Don’t know* | 2(3.3) | 1(8.3) | 0 | 1(7.7) | 0 |  |
|  | *N/A* | 2(3.3) | 0 | 2(13.3) | 0 | 0 |  |
|  | **Median**^b^ |  | 3 | 4 | 3 | 2.5 |  |
| 1. *Government or Political support* | *Agree* | 13(21.3) | 3(25.0) | 3(20.0) | 3(21.4) | 4(20) | *0.6506* |
|  | *Neutral* | 14(23.0) | 1(8.3) | 7(46.7) | 3(21.4) | 3(15) |  |
|  | *Disagree* | 30(49.2) | 6(50.0) | 4(26.6) | 8(57.1) | 12(60) |  |
|  | *Don’t know* | 1(1.6) | 1(8.3) | 0 | 1(7.1) | 0 |  |
|  | *N/A* | 3(4.9) | 1(8.3) | 1(6.7) | 0 | 1(5) |  |
|  | **Median**^b^ |  | 2 | 3 | 2 | 2 |  |
| 1. *Non-government or private sector support* | *Agree* | 20(33.9) | 5(41.6) | 4(28.6) | 5(35.7) | 6(31.6) | *0.8971* |
|  | *Neutral* | 17(28.8) | 0 | 5(35.7) | 5(35.7) | 7(36.8) |  |
|  | *Disagree* | 18(30.5) | 6(50.0) | 4(28.6) | 2(21.4) | 5(26.3) |  |
|  | *Don’t know* | 2(3.4) | 1(8.3) | 0 | 1(7.1) | 0 |  |
|  | *N/A* | 2(3.4) | 0 | 1(7.1) | 0 | 1(5.3) |  |
|  | **Median**^b^ |  | 2 | 3 | 3 | 3 |  |
| 1. *Other resources (e.g., time)* | *Agree* | 15(25) | 4(33.3) | 4(26.7) | 4(30.8) | 3(15) | *0.8342* |
|  | *Neutral* | 19(31.7) | 4(33.3) | 5(33.3) | 3(23.1) | 7(35) |  |
|  | *Disagree* | 19(31.7) | 4(33.4) | 4(26.7) | 2(30.8) | 7(35) |  |
|  | *Don’t know* | 5(8.3) | 0 | 0 | 2(15.4) | 3(15) |  |
|  | *N/A* | 2(3.3) | 0 | 2(13.3) | 0 | 0 |  |
|  | **Median**^b^ |  | 3 | 3 | 3 | 3 |  |

^a^Responses of strongly agree and agree are summed and displayed under the scale “agree”. Likewise, responses of strongly disagree and disagree are summed and displayed under the scale “disagree”. ^b^Median values reported reflect the 5-point Likert scale ranging from strongly disagree (1) to strongly agree (5). The response of “Don’t Know” and “N/A” were considered missing.

Table S3: Summary of motivations for integrating health into community design, stratified by job function.

| **Survey Question:** *To what extent do you agree that the following motivates or drives you to consider healthy living in decision-making about community and/or building design in your position?* | *Response scales^a^* | **All professionals**  n (%) | **Public Health professionals**  n (%) | **Planning professionals**  n (%) | **Policy/program development professionals**  n (%) | **Other professionals**  n (%) | *Kruskal-Wallis Test*  *p-value* |
| --- | --- | --- | --- | --- | --- | --- | --- |
| 1. *Interest in societal or community impacts (e.g. supporting local economies, sustainability, and health)* | *Agree* | 60(98.4) | 12(100) | 15(100) | 14(100) | 19(95) | *0.4767* |
|  | *Neutral* | 0 | 0 | 0 | 0 | 0 |  |
|  | *Disagree* | 0 | 0 | 0 | 0 | 0 |  |
|  | *Don’t know* | 1(1.6) | 0 | 0 | 0 | 1(5) |  |
|  | *N/A* | 0 | 0 | 0 | 0 | 0 |  |
|  | **Median**^b^ |  | 5 | 5 | 4.5 | 5 |  |
| 1. *Legislation, regulations, and codes* | *Agree* | 25(41) | 3(25.0) | 12(80) | 2(14.3) | 8(40) | *0.0131^**^* |
|  | *Neutral* | 13(21.3) | 4(33.3) | 2(13.3) | 3(21.4) | 4(20) |  |
|  | *Disagree* | 18(29.5) | 4(33.3) | 1(6.7) | 6(42.9) | 7(35.0) |  |
|  | *Don’t know* | 4(6.6) | 1(8.3) | 0 | 2(14.3) | 1(5) |  |
|  | *N/A* | 1(1.6) | 0 | 0 | 1(7.1) | 0 |  |
|  | **Median**^b^ |  | 3 | 4 | 2 | 3 |  |
| 1. *Standards* | *Agree* | 29(47.5) | 6(50.0) | 10(66.7) | 4(28.6) | 9(45) | *0.3079* |
|  | *Neutral* | 13(21.3) | 3(25.0) | 4(26.7) | 3(21.4) | 3(15) |  |
|  | *Disagree* | 13(21.3) | 2(16.7) | 1(6.7) | 3(21.4) | 7(35) |  |
|  | *Don’t know* | 5(8.2) | 1(8.3) | 0 | 3(21.4) | 1(5) |  |
|  | *N/A* | 1(1.6) | 0 | 0 | 1(7.1) | 0 |  |
|  | **Median**^b^ |  | 4 | 4 | 3 | 3 |  |
| 1. *Guidelines* | *Agree* | 33(55) | 7(63.7) | 10(66.7) | 3(21.4) | 13(65) | *0.1176* |
|  | *Neutral* | 13(21.7) | 2(18.2) | 5(33.3) | 4(28.6) | 2(10) |  |
|  | *Disagree* | 8(13.3) | 1(9.1) | 0 | 3(21.4) | 4(20) |  |
|  | *Don’t know* | 5(8.3) | 1(9.1) | 0 | 3(21.4) | 1(5) |  |
|  | *N/A* | 1(1.7) | 0 | 0 | 1(7.1) | 0 |  |
|  | **Median**^b^ |  | 4 | 4 | 3 | 4 |  |
| 1. *Healthy community and/or building certification systems or criteria* | *Agree* | 28(45.9) | 6(50.0) | 7(46.7) | 4(28.6) | 11(55) | *0.6711* |
|  | *Neutral* | 15(24.6) | 3(25.0) | 6(40) | 1(7.1) | 5(25) |  |
|  | *Disagree* | 8(13.1) | 2(16.7) | 1(6.7) | 4(28.6) | 1(5) |  |
|  | *Don’t know* | 7(11.5) | 1(8.3) | 1(6.7) | 3(21.4) | 2(5) |  |
|  | *N/A* | 3(4.9) | 0 | 0 | 2(14.3) | 1(5) |  |
|  | **Median**^b^ |  | 4 | 3.5 | 3 | 4 |  |
| 1. *Client interests and/or values* | *Agree* | 42(68.9) | 8(66.7) | 10(46.7) | 11(78.6) | 13(65) | *0.8710* |
|  | *Neutral* | 6(9.8) | 1(8.3) | 2(13.3) | 0 | 3(15) |  |
|  | *Disagree* | 6(9.8) | 1(8.3) | 2(13.3) | 1(7.1) | 2(10) |  |
|  | *Don’t know* | 4(6.6) | 2(16.7) | 0 | 1(7.1) | 1(5) |  |
|  | *N/A* | 3(4.9) | 0 | 1(6.7) | 1(7.1) | 1(5) |  |
|  | **Median**^b^ |  | 4 | 4 | 4 | 4 |  |
| 1. *Organizational interest and/or values* | *Agree* | 46(75.4) | 10(63.3) | 10(66.7) | 11(78.6) | 15(75) | *0.6458* |
|  | *Neutral* | 8(13.1) | 0 | 3(20) | 2(14.3) | 3(15) |  |
|  | *Disagree* | 3(4.9) | 1(8.3) | 1(6.7) | 1(7.1) | 0 |  |
|  | *Don’t know* | 2(3.3) | 1(8.3) | 0 | 0 | 1(5) |  |
|  | *N/A* | 2(3.3) | 0 | 1(6.7) | 0 | 1(5) |  |
|  | **Median**^b^ |  | 4 | 4 | 4 | 4 |  |
| 1. *Personal interest and/or values* | *Agree* | 56(91.8) | 12(100) | 12(80) | 13(92.9) | 19(95.0) | *0.0072^**^* |
|  | *Neutral* | 3(4.9) | 0 | 2(13.3) | 1(7.1) | 0 |  |
|  | *Disagree* | 0 | 0 | 0 | 0 | 0 |  |
|  | *Don’t know* | 2(3.3) | 0 | 1(6.7) | 0 | 1(5) |  |
|  | *N/A* | 0 | 0 | 0 | 0 | 0 |  |
|  | **Median**^b^ |  | 5 | 4 | 4 | 5 |  |
| 1. *Public awareness of the impact of community and/or building design on healthy living* | *Agree* | 43(70.5) | 6(50.0) | 10(66.7) | 9(64.3) | 14(70) | 0.1256 |
|  | *Neutral* | 6(9.8) | 5(41.7) | 3(20) | 1(7.1) | 2(10) |  |
|  | *Disagree* | 8(13.1) | 1(8.3) | 2(13.3) | 3(21.4) | 2(10) |  |
|  | *Don’t know* | 3(4.9) | 0 | 0 | 1(7.1) | 1(5) |  |
|  | *N/A* | 1(1.6) | 0 | 0 | 0 | 1(5) |  |
|  | **Median**^b^ |  | 4 | 4 | 4 | 4 |  |
| 1. *Ability to sustain improved community and/or building design over the long term* | *Agree* | 49(80.3) | 9(75.0) | 14(93.3) | 10(71.4) | 16(80) | *0.6183* |
|  | *Neutral* | 3(4.9) | 2(16.7) | 0 | 0 | 1(5) |  |
|  | *Disagree* | 4(6.6) | 0 | 1(6.7) | 1(7.1) | 2(10) |  |
|  | *Don’t know* | 5(8.2) | 1(8.3) | 0 | 3(21.4) | 1(5) |  |
|  | *N/A* | 0 | 0 | 0 | 0 | 0 |  |
|  | **Median**^b^ |  | 4 | 4 | 4 | 5 |  |
| 1. *Government sector healthy community and/or building initiatives* | *Agree* | 34(55.7) | 8(66.7) | 8(53.3) | 8(57.1) | 10(50) | *0.6222* |
|  | *Neutral* | 11(18.0) | 2(16.7) | 3(20) | 4(28.6) | 2(10) |  |
|  | *Disagree* | 10(16.4) | 1(8.3) | 3(20) | 1(7.1) | 5(25) |  |
|  | *Don’t know* | 5(8.2) | 1(8.3) | 1(6.7) | 1(7.1) | 2(10) |  |
|  | *N/A* | 1(1.6) | 0 | 0 | 0 | 1(5) |  |
|  | **Median**^b^ |  | 4 | 4 | 4 | 4 |  |
| 1. *Non-government or private sector healthy community and/or building initiatives* | *Agree* | 37(60.7) | 8(66.6) | 9(60) | 9(64.3) | 11(55) | *0.6056* |
|  | *Neutral* | 10(16.4) | 1(8.3) | 2(13.3) | 4(28.6) | 3(15) |  |
|  | *Disagree* | 8(13.1) | 2(16.7) | 3(20) | 0 | 3(15) |  |
|  | *Don’t know* | 5(8.2) | 1(8.3) | 1(6.7) | 1(7.1) | 2(10) |  |
|  | *N/A* | 1(1.6) | 0 | 0 | 0 | 1(5) |  |
|  | **Median**^b^ |  | 4 | 4 | 4 | 4 |  |
| 1. *Mindfulness of assets and reputation* | *Agree* | 31(50.8) | 7(58.3) | 6(40) | 7(50) | 11(55) | *0.5802* |
|  | *Neutral* | 14(23.0) | 3(25.0) | 3(20) | 3(21.4) | 5(25) |  |
|  | *Disagree* | 8(13.1) | 1(8.3) | 4(26.7) | 1(7.1) | 2(10) |  |
|  | *Don’t know* | 6(9.8) | 1(8.3) | 1(6.7) | 3(21.4) | 1(5) |  |
|  | *N/A* | 2(3.3) | 0 | 1(6.7) | 0 | 1(5) |  |
|  | **Median^b^** |  | 4 | 3 | 4 | 4 |  |
| 1. *Scientific evidence of the health impacts of community and/or building design* | *Agree* | 44(73.3) | 10(90.9) | 9(60) | 9(64.3) | 16(80) | *0.0273^**^* |
|  | *Neutral* | 5(8.3) | 0 | 3(20) | 1(7.1) | 1(5) |  |
|  | *Disagree* | 7(11.7) | 0 | 2(13.3) | 3(21.4) | 2(10.0) |  |
|  | *Don’t know* | 4(6.7) | 1(9.1) | 1(6.7) | 1(7.1) | 1(5) |  |
|  | *N/A* | 0 | 0 | 0 | 0 | 0 |  |
|  | **Median**^b^ |  | 5 | 4 | 4 | 5 |  |
| 1. *Collaboration and communication across project partners and sectors* | *Agree* | 44(72.1) | 8(66.7) | 10(66.7) | 9(64.3) | 17(85) | *0.1720* |
|  | *Neutral* | 8(13.1) | 2(16.7) | 3(20) | 3(21.4) | 0 |  |
|  | *Disagree* | 3(4.9) | 0 | 1(6.7) | 1(7.1) | 1(5) |  |
|  | *Don’t know* | 5(8.2) | 2(16.7) | 1(6.7) | 1(7.1) | 1(5) |  |
|  | *N/A* | 1(1.6) | 0 | 0 | 0 | 1(5) |  |
|  | **Median**^b^ |  | 4.5 | 4 | 4 | 4 |  |
| 1. *Community support* | *Agree* | 45(73.8) | 10(83.4) | 10(66.7) | 12(85.7) | 13(65) | *0.4386* |
|  | *Neutral* | 10(16.4) | 1(8.3) | 3(20) | 2(14.3) | 4(20) |  |
|  | *Disagree* | 2(3.3) | 0 | 1(6.7) | 0 | 1(5) |  |
|  | *Don’t know* | 3(4.9) | 1(8.3) | 1(6.7) | 0 | 1(5) |  |
|  | *N/A* | 1(1.6) | 0 | 0 | 0 | 1(5) |  |
|  | **Median**^b^ |  | 4 | 4 | 4 | 4 |  |
| 1. *Government sector support* | *Agree* | 32(52.5) | 7(58.3) | 7(46.7) | 10(71.4) | 8(40) | *0.7579* |
|  | *Neutral* | 16(26.2) | 2(16.7) | 6(40) | 3(21.4) | 5(25) |  |
|  | *Disagree* | 9(14.8) | 2(16.7) | 2(13.3) | 0 | 5(25) |  |
|  | *Don’t know* | 3(4.9) | 1(8.3) | 0 | 1(7.1) | 1(5) |  |
|  | *N/A* | 1(1.6) | 0 | 0 | 0 | 1(5) |  |
|  | **Median**^b^ |  | 4 | 3 | 4 | 3 |  |
| 1. *Non-Government or private sector support* | *Agree* | 38(62.3) | 7(58.3) | 10(66.7) | 9(64.3) | 12(60) | *0.9365* |
|  | *Neutral* | 14(23.0) | 2(16.7) | 4(26.7) | 4(28.6) | 4(20) |  |
|  | *Disagree* | 5(8.2) | 2(16.7) | 1(6.7) | 0 | 2(10) |  |
|  | *Don’t know* | 3(4.9) | 1(8.3) | 0 | 1(7.1) | 1(5) |  |
|  | *N/A* | 1(1.6) | 0 | 0 | 0 | 1(5) |  |
|  | **Median**^b^ |  | 4 | 4 | 4 | 4 |  |

^a^Responses of strongly agree and agree are summed and displayed under the scale “agree”. Likewise, responses of strongly disagree and disagree are summed and displayed under the scale “disagree”. ^b^Median values reported reflect the 5-point Likert scale ranging from strongly disagree (1) to strongly agree (5). The response of “Don’t Know” and “N/A” were considered missing. ^**^p<0.05

Table S4: Summary of healthy built environment practices separated by job functions.

| **Survey Question:** *How often do you do the following in your position to ensure healthy living is considered in decision-making about community and/or building design?* | *Response scales^a^* | **All professionals**  n (%) | **Public Health professionals**  n (%) | **Planning professionals**  n (%) | **Policy/program development professionals**  n (%) | **Other professionals**  n (%) | *Kruskal-Wallis Test*  *p-values* |
| --- | --- | --- | --- | --- | --- | --- | --- |
| *1. Providing scientific evidence on the impact of community and/or building design on healthy living to stakeholders* | *Frequently* | 18(30) | 4(33.4) | 3(21.4) | 5(35.7) | 6(30) | *0.8797* |
|  | *Occasionally/Sometimes* | 17(28.3) | 4(33.3) | 5(35.7) | 2(14.3) | 6(30) |  |
|  | *Rarely* | 20(33.3) | 4(33.3) | 6(42.9) | 5(35.7) | 5(25) |  |
|  | *N/A* | 5(8.3) | 0 | 0 | 2(14.3) | 3(15) |  |
|  | **Median**^b^ |  | 3 | 3 | 3 | 3 |  |
| *2) Engaging with stakeholders and end-users such as clients or community residents at an early stage of community and/or building design about including healthy features* | *Frequently* | 20(33.9) | 2(16.7) | 4(28.6) | 6(42.9) | 8(42.1) | *0.3501* |
|  | *Occasionally/Sometimes* | 19(32.2) | 6(50.0) | 5(35.7) | 3(21.4) | 5(26.3) |  |
|  | *Rarely* | 17(28.8) | 4(33.4) | 5(35.7) | 4(28.6) | 4(21.1) |  |
|  | *N/A* | 3(5.1) | 0 | 0 | 1(7.1) | 2(10.5) |  |
|  | **Median**^b^ |  | 3 | 3 | 3 | 3 |  |
| *3. Advocating for the inclusion of healthy features into community and/or building design* | *Frequently* | 28(48.3) | 3(27.3) | 8(57.1) | 7(50) | 10(52.7) | *0.3095* |
|  | *Occasionally/Sometimes* | 18(31.0) | 5(45.5) | 4(28.6) | 4(28.6) | 5(26.3) |  |
|  | *Rarely* | 10(17.2) | 3(27.3) | 2(14.3) | 2(14.3) | 3(15.8) |  |
|  | *N/A* | 2(3.5) | 0 | 0 | 1(7.1) | 1(5.3) |  |
|  | **Median**^b^ |  | 3 | 4 | 4 | 4 |  |
| *4. Taking responsibility for the inclusion of healthy features into community and/or building design* | *Frequently* | 18(30.5) | 0 | 5(35.7) | 4(28.6) | 9(47.4) | *0.0067^**^* |
|  | *Occasionally/Sometimes* | 15(25.4) | 2(16.7) | 5(35.7) | 4(28.6) | 4(21.1) |  |
|  | *Rarely* | 22(37.3) | 9(75.0) | 3(21.4) | 5(35.7) | 5(26.3) |  |
|  | *N/A* | 4(6.8) | 1(8.3) | 1(7.1) | 1(7.1) | 1(5.3) |  |
|  | **Median**^b^ |  | 2 | 3 | 3 | 3.5 |  |
| *5. Collaborating and sharing knowledge with partners around implementing healthy community and/or building design features* | *Frequently* | 32(54.2) | 5(41.6) | 6(42.9) | 9(64.3) | 12(63.2) | *0.5286* |
|  | *Occasionally/Sometimes* | 15(25.4) | 4(33.3) | 6(42.9) | 2(14.3) | 3(15.8) |  |
|  | *Rarely* | 10(16.9) | 3(25.0) | 2(14.3) | 2(14.3) | 3(25.8) |  |
|  | *N/A* | 2(3.4) | 0 | 0 | 1(7.1) | 1(5.3) |  |
|  | **Median**^b^ |  | 3 | 3 | 4 | 4 |  |
| *6. Evaluating the degree of implementation of healthy community and/or building design* | *Frequently* | 15(25.4) | 2(16.7) | 4(28.6) | 4(28.6) | 5(26.3) | *0.0473^**^* |
|  | *Occasionally/Sometimes* | 15(25.4) | 0 | 5(35.7) | 6(42.9) | 4(21.1) |  |
|  | *Rarely* | 26(44.1) | 10(83.4) | 5(35.7) | 3(21.4) | 8(42.1) |  |
|  | *N/A* | 3(5.1) | 0 | 0 | 1(7.1) | 2(10.5) |  |
|  | **Median**^b^ |  | 2 | 3 | 3 | 3 |  |
| *7. Evaluating the impacts of implementation of healthy community and/or building design* | *Frequently* | 12(20.3) | 1(8.3) | 4(28.6) | 3(21.4) | 4(21.1) | *0.0923* |
|  | *Occasionally/Sometimes* | 16(27.1) | 1(8.3) | 3(21.4) | 7(50) | 5(26.3) |  |
|  | *Rarely* | 28(47.5) | 10(83.4) | 7(50) | 3(21.4) | 8(42.1) |  |
|  | *N/A* | 3(5.1) | 0 | 0 | 1(7.1) | 2(10.5) |  |
|  | **Median**^b^ |  | 2 | 2.5 | 3 | 3 |  |

^a^Responses of frequently and always are summed and displayed under the scale “frequently”. Likewise, responses of never and rarely are summed and displayed under the scale “rarely”. ^b^Median values reported reflect the 5-point Likert scale ranging from never (1) to Always (5). The response of “N/A” was considered missing. ^**^p<0.05
